# The use of distortion product otoacoustic emissions (DPOAE) records to estimate effect of vitamin B complex on changing severity of tinnitus

**DOI:** 10.1016/j.amsu.2018.10.035

**Published:** 2018-11-05

**Authors:** Husam Majeed Hameed, Alaa Husain Eleue, Ahmed Mohammed Taqi Al Mosawi

**Affiliations:** a AL-Karama Teaching Hospital, College of Medicine, Wasit University, Iraq; b ENT Department, AL-Karama Teaching Hospital, Iraq

**Keywords:** B-complex deficiency, OAE, DPOAE, Tinnitus, SNHL

## Abstract

**Introduction:**

Patients that are complaining from tinnitus but have normal hearing comprise an uncommon group and there is rare literature about them. Deficiency in B-complex vitamins has been shown to result in tinnitus and supplementation may improve the symptom which means manipulation of cochlear status. Distortion Product Otoacoustic emissions (DPOAEs) used frequently to assess cochlear status.

**Objectives:**

The uses of DPOAE changing amplitude as a parameter to estimate effect of vitamin B complex on changing the severity of tinnitus in patients with tinnitus only in comparison to patients have tinnitus with Sensory Neural Hearing Loss (SNHL) and a control group.

**Methods:**

Prospective observational cohort study was performed in AL-Karama Teaching Hospital as a secondary medical care center, from 01/01/2012 to 31/12/2016. A three groups had been evaluated clinically and by OAE device (ECHOLAB) to study the DPOAE changing amplitude before and after one month of supplementation of B-complex vitamins to 25 patients in study group (1) have (tinnitus only) and also to 25 patients in study group (2) have (Tinnitus + SNHL) but leaving control group who were a 25 medical staff (No Tinnitus and nor SNHL) without treatment.

**Results:**

It has been found that among Study group 1 (patients had Tinnitus only), Study group 2 (patients had Tinnitus + SNHL) and the control group: 16 patients (44%), P-value = 0.000 HS; 28 patients (78%) P-value = 0.000 HS; and 0% respectively, they had low Amplitude of DPOAE recorded by OAE device (ECHOLAB). After one month from treatment with Neurobine ampules for study group 1& 2 but not control group (whom receive no treatment), those who got clinical improvement + increase Amplitude of DPOAE (i.e. subjective + objective changes) after treatment were 10 patients (28%) P-value = 0.000 HS in study group1, and two patients (5%) P-value 0.321 NS in study group 2, and there was no changes (zero) among control group.

**Conclusions:**

The uses of DPOAE changing amplitude could be used as a parameter to estimate effect of vitamin B complex on changing the severity of Tinnitus in patients with tinnitus only or those have Tinnitus with SNHL. The supplementation of vitamin B complex could improve the tinnitus severity especially in patients with tinnitus without SNHL.

## Highlights

• The uses of DPOAE changing amplitude could be used as a parameter to estimate effect of Vitamin B complex on changing the severity of Tinnitus.
• Vitamin B complex deficiency could be factor increase the severity of tinnitus.
• Vitamin B complex its supplement could improve the tinnitus severity especially in patients with tinnitus without SNHL.
• As recommendation: a routine B-complex vitamins serum levels should be determined when evaluating patients for tinnitus and those with SNHL.

## 1 Introduction

Tinnitus is not a solitary or distinct disease, but it is a symptom of many pathologies. Sometimes in one patient, numerous pathological mechanisms may coincide. 15–35% of patients with Tinnitus presented to the audiological units with normal hearing [1].

Grading of severity of tinnitus is quantifying according to the new classifying system that measures the severity of tinnitus in adults which has been suggested by the British Association of Otolaryngologists, Head and Neck Surgeons (BAOL-HNS), which arise from grad I(slight: Only perceived in silent environment, very easily masked, not interfere with sleep or daily events.) to Grade 5(catastrophic: All tinnitus symptoms at a worse level and accompanying with psychological pathology that can be found in hospital or GP records [2].

A strong etiopathology of tinnitus is not completely set, multifactorial theory is suggested but there is enough evidence to suggest that” tinnitus is the result of an abnormal spontaneous neural activity within the auditory system, which varies from the standard, non-stimulus state [3]. A foundation of modification in spontaneous activity within the auditory system could be an injury or abnormal task at any level, from the cochlea to the highest levels of the auditory system [3]. It had been also founded that there is an association between tinnitus and reduced outer hair cells activity which indicate the outer hair cells of cochlea are involved in the generation of tinnitus [4].

It can be assumed that the alteration in spontaneous activity, leading to tinnitus, arises from changes in the balance between excitation and inhibition within the auditory system, through different underlying mechanisms [5].

The most common presenting symptom that led a patient with Noise Induced Hearing loss(NIHL) was found to be the tinnitus rather than the hearing difficulty, and this can be explained on the basic knowledge that NIHL usually involve the high frequencies at first, which is usually away from the speech frequencies. hence the patient can develop the NIHL and they did not complain from hearing loss and may only has ear discomfort or tinnitus which could be the presenting symptoms [6].

The B-complex vitamins are a main constituent of enzyme system that control protein, e.g. Cyanocobalamin (vitamin B12) and thiamine hydrochloride (vitamin B1) work as enzymes while pyridoxine hydrochloride (vitamin B6) works as co-enzyme, hence the B-complex vitamins family of nutrients have been assembled together because of the conjoined relationship in their purpose within human enzyme systems, and their spreading in ordinary diet sources. Lack in these vitamins has been revealed to consequence in tinnitus and addition to diet may ameliorate the symptom [7]. The B vitamins are water soluble and can be absorbed easily, except vitamin B12, whose it's absorption can be achieved and empowered by intramuscular root [8].

Vitamin B1 (thiamine) is a nutrient with an important effect in serving a healthy central nervous system. Some patients have visualize that supplementation of vitamin B1 treat the tinnitus they complain from it [9]. There may be some correlation between the decline in vitamin B12 levels and the increasing prevalence of tinnitus in the elderly [10,11].

In humans, vitamin B12 deficiency may affect haematological, gastrointestinal and neurological systems. Neurological damage is the result of pathological changes, which may at the end result in demyelination, axonal degeneration and death of the neurons [12,13].

Studies that had been done on an Electrophysiological basis in patients with vitamin B12 deficiency demonstrated the sensory motor axonopathy due to neurological lesions such as myelopathy, myeloneuropathy, peripheral neuropathy, optic neuropathy and axonal demyelinating [14].

The estimation and evaluation of (B vitamins) levels in body is so complicated, hence the levels of (B vitamins) are considered normal in dispute, and disruption possible at numerous levels, so from this point of view; many physicians have come to the conclusion that the only reliable method to estimate the effects of superfine B12 deficiency on the blood, especially in elderly age group of patients, is to clearly observe changes (especially psychological changes) post-therapy and to determine empirically the best amount of supplementation for the every particular patient. Since B12 is not toxic, except perhaps at extremely high levels, the empirical prescribing and usage of relatively large doses to treat patients will have no danger and it will still safe supplement [15].

Coelho C et al. concluded that a use of dietary supplements to treat tinnitus is common, particularly with Ginkgo biloba, lipoflavonoids, magnesium, melatonin, vitamin B12, and zinc. It is likely that some supplements will help with sleep for some patients [16]. But this came in contrary to what had been concluded by Polanski JF et al. who founded that there was no benefit from the use of antioxidant agents(Ginkgo biloba dry extract, α-lipoic acid vitamin C, papaverine hydrochloride vitamin E) for tinnitus in his sample [17].

An audiometric assessment should be done to All patients with tinnitus, since the subjective complaint from the patients usually are loosely associated with real acoustic properties [18].

The tools of diagnosis and testing should include audiometry, speech discrimination testing, and tympanometry [19].

Otoacoustic emissions (OAEs), which is described by Kemp in 1978, increased hopes for a better understanding of tinnitus, as they directly evaluate the cochlea, especially the external hair cells. The primary purpose of otoacoustic emission test is to determine cochlear status, especially hair cell functioning [20].

However, initial tests using spontaneous OAEs were frustrating, and the studies performed with transient OAEs have not reached a consensus. Thus, distortion product OAEs (DPOAEs) would be convenient for this purpose, because they permit the analysis of a larger spectrum of frequencies, providing a segmented analysis of practically all of the extension of the cochlea [21].

Distortion product otoacoustic emission (DPOAE) is a type of OAE in which the stimulus consists of two different pure tones of two different frequencies (i.e., F1 and F2; F2 >F1) and two intensity levels (i.e., L1 and L2; L1 >L2) the relationship between L1/L2 and F1/F2 dictates the frequency response. At higher frequencies the DPOAEs can be reliably and better recorded than transient evoked OAE. Since that property, it is now better to demonstrate cochlear damage more clearly through DPOAEs recordings [22].

Patients with tinnitus and normal hearing constitute an uncommon group and there is rare literature on tinnitus cases with normal hearing [4]. On the other hand there are many literature discussed tinnitus in patients with SNHL, like Sermin Kibar et al. whom stated that “it was observed that simultaneously administration of vitamin B12 during noise had no protective effect on permanent threshold shift^”^[23].

From all the above we can see there were many attempts to find the correlation between dietary supplements, vitamin B complex and to treatment of tinnitus from one side and the uses of OAE as diagnostic tool for tinnitus in patients. Hence the aim of this study is about the use of DPOAE changing amplitude as a parameter to estimate effect of vitamin B complex on changing the severity of tinnitus in patients with tinnitus only in comparison to patients have tinnitus with SNHL and people within control group.

## 2 Methods

The method used is a prospective observational cohort study analyseas outcomes of a two groups of patients who were transferred because of hearing problem to Audiology unit in AL-Karama Teaching Hospital as a secondary medical care center, from 01/01/2012 to 30/12/2016. Before that the study had been approved by the Ethical Approval Committee of the College of Medicine Wasit University with the No. 34/W256.

• During a period of five years we selected all the 25 patients (Study group 1) with tinnitus only i.e. (normal hearing), 19 bilateral tinnitus+6 unilateral, hence (n1) = 44 ears.

Patients included in this study were with no history of ototoxic drug intake, no exposure to noise/acoustic trauma or any history of ear surgery, not diabetic nor hypertensive patients and not pregnant women. Also exclusion of patients who had middle ear disorders which can result in conductive hearing loss).

• Another 25 patients have been Selected (Study group 2) (19 bilateral tinnitus+6 unilateral) with tinnitus + SNHL and match in age & gender to study group I., hence (n2) = 44 ears.

*NB. Because the limited number of patients with tinnitus only i.e. (normal hearing) we had selected all 25 patients visited our outpatient clinic over 5 years (excluding those not fit our criteria) while we had selected first 25 patients with tinnitus + SNHL visited our outpatient clinic over 3years because there are large number of patients within this category (excluding those not fit our criteria).

Both study group 1& 2 had been classified demographically in to age group categories, sex categories and whether they are living in urban or rural areas, hence the control group had been selected to match them accordingly.

Control group: was 25 medical staff in our hospital (n3 = 50 ears) who have no Tinnitus and no SNHL and match in age & gender to study groups that mean all were otologically normal without tinnitus; never had any ear infection; nor exposed to noise or had any ear surgery.

Design of the Study was that all participants were informed about the purposes of the study and to its protocol and they can accept or refuse to take part in this study, an informed consent were taken. They answered a questionnaire, which include questions relating to their name, age, full medical history, ENT & medications history. Then full ENT examinations done. Grading of severity of tinnitus according to grading system that assesses the severity of tinnitus in adults which has been proposed by the British Association of Otolaryngologists, Head and Neck Surgeons (BAOL-HNS), which start from grad I (slight) to Grade 5(catastrophic).

### 2.1 Clinical assessment

1.Otoscopic examination: by using the auroscope, assessment of the external and middle ear were carried out on each ear of all patients by the Ear Nose and Throat Consultant (ENT doctor) to assess any disorders of all patients by otoscopic examination. We had excluded patients who had any detectable middle ear diseases like (otitis media with effusion, perforated tympanic membrane, Otosclerosis or any other ear lesions which may led to conductive type of hearing impairment)

Hence patients included in this study were limited to only patients who had normal otoscopic examination in both study groups and control group.

2. Pure tone audiometery(PTA): detection of hearing threshold levels in the scope of 500–8000Hz were done on each ear of the all patients using descending of 10 dB step size and 5 dB step size to decide the lowest hearing threshold levels at all tested frequencies. All measurements were conceded out by an experienced audiologist in an isolated test sound prove booth using clinical diagnostic audiometer (Audio Traveller AA 220, Inter-acoustics A/S).

**Fig. 1:**
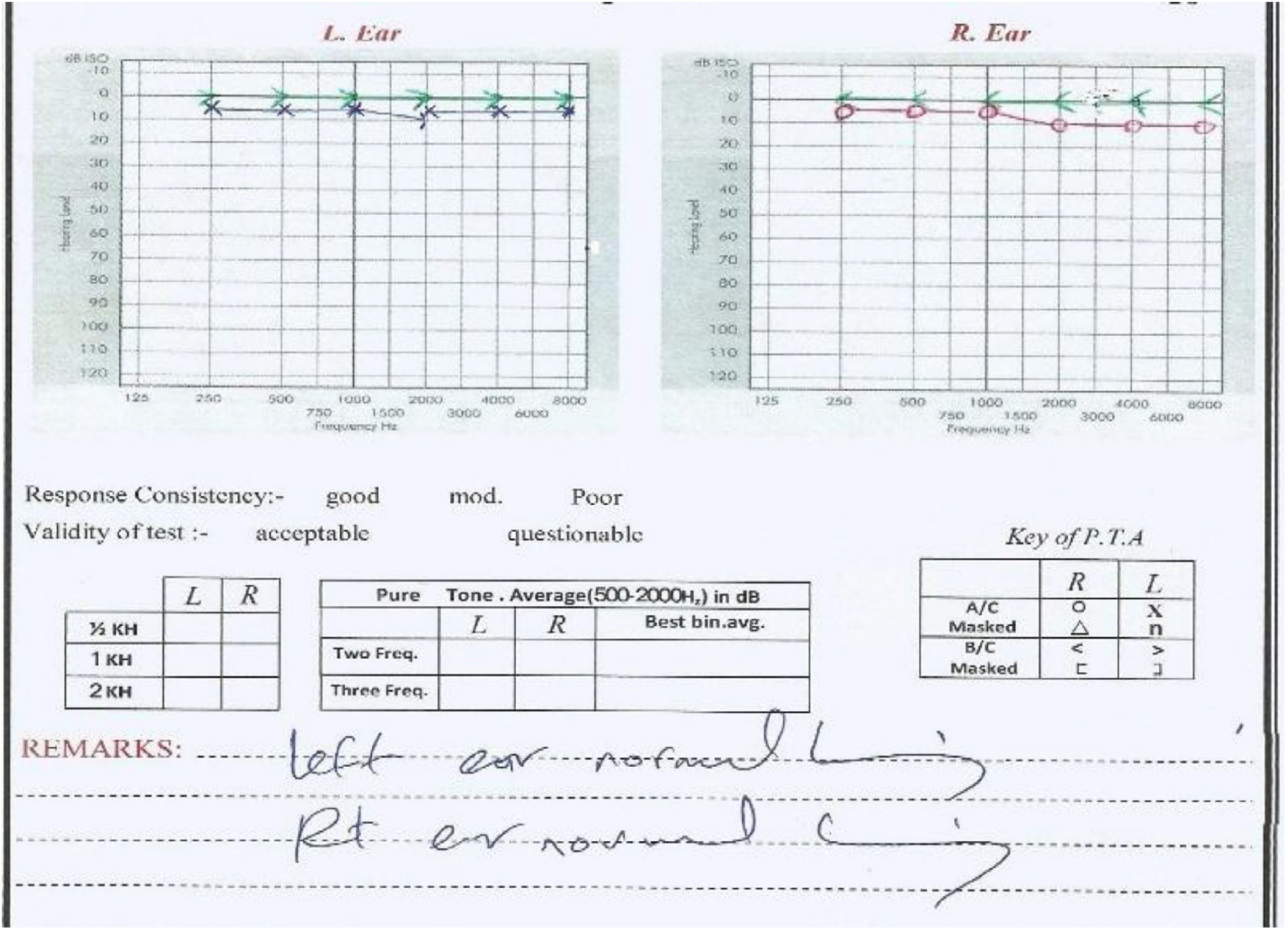
The Normal Pure Tone Audiogram (PTA) for a patient with tinnitus without SNHL.

Normal hearing threshold levels were a must (Fig. 1) in All patients of study group1 (Tinnitus only); while a different hearing loss over many frequencies were accepted in the Study goup2 (Tinnitus + SNHL) and again a normal hearing threshold levels were a must in all patients within the control group who were otologically normal without tinnitus; never had any ear infection; nor exposed to noise or had any ear surgery.

This control group underwent all assessments carried out on both study groups including otoscopic examination, pure tone audiometry, tympanometry and distortion product otoacoustic emissions, but they did not receive Neurobine Ampules.

**Fig. 2:**
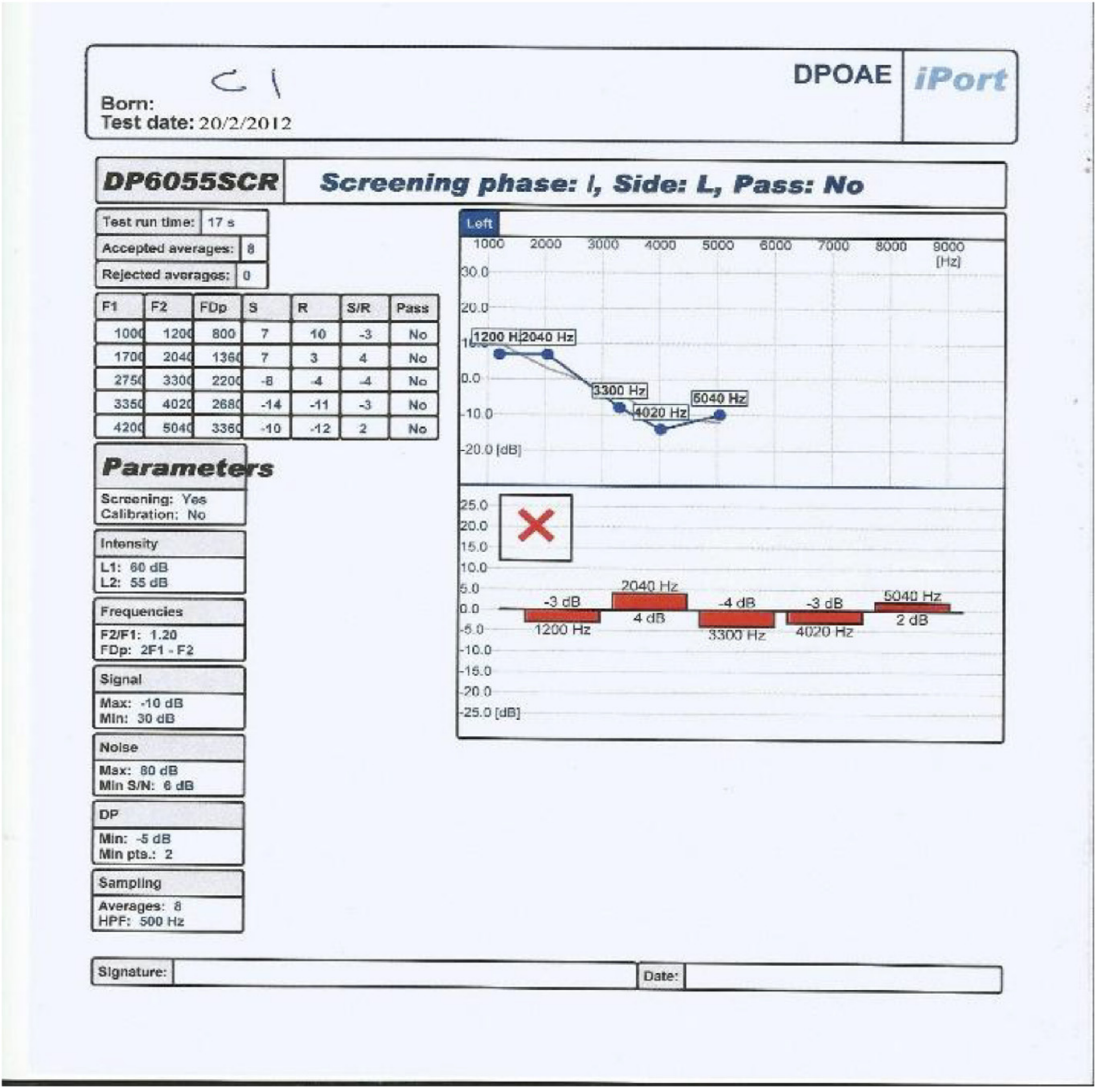
DPOAE of left ear before treatment.

**Fig. 3:**
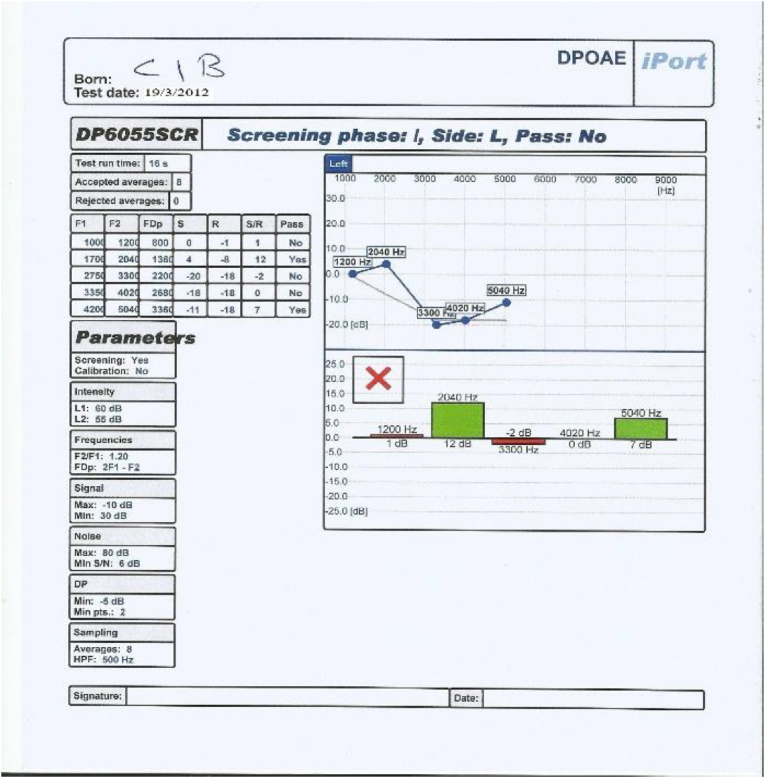
DPOAE of left ear after treatment.

**Fig. 4:**
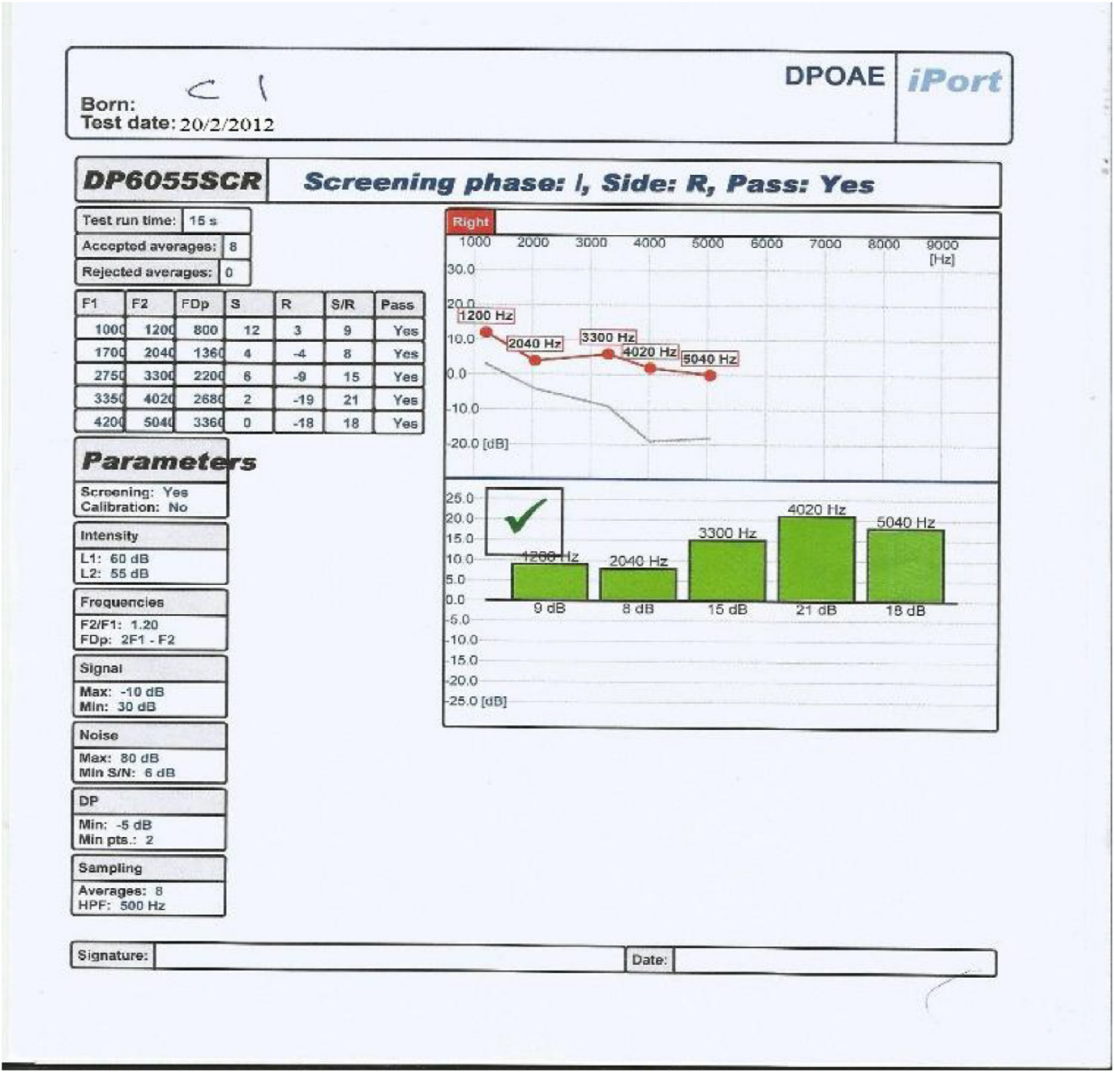
DPOAE of right ear before treatment.

**Fig. 5:**
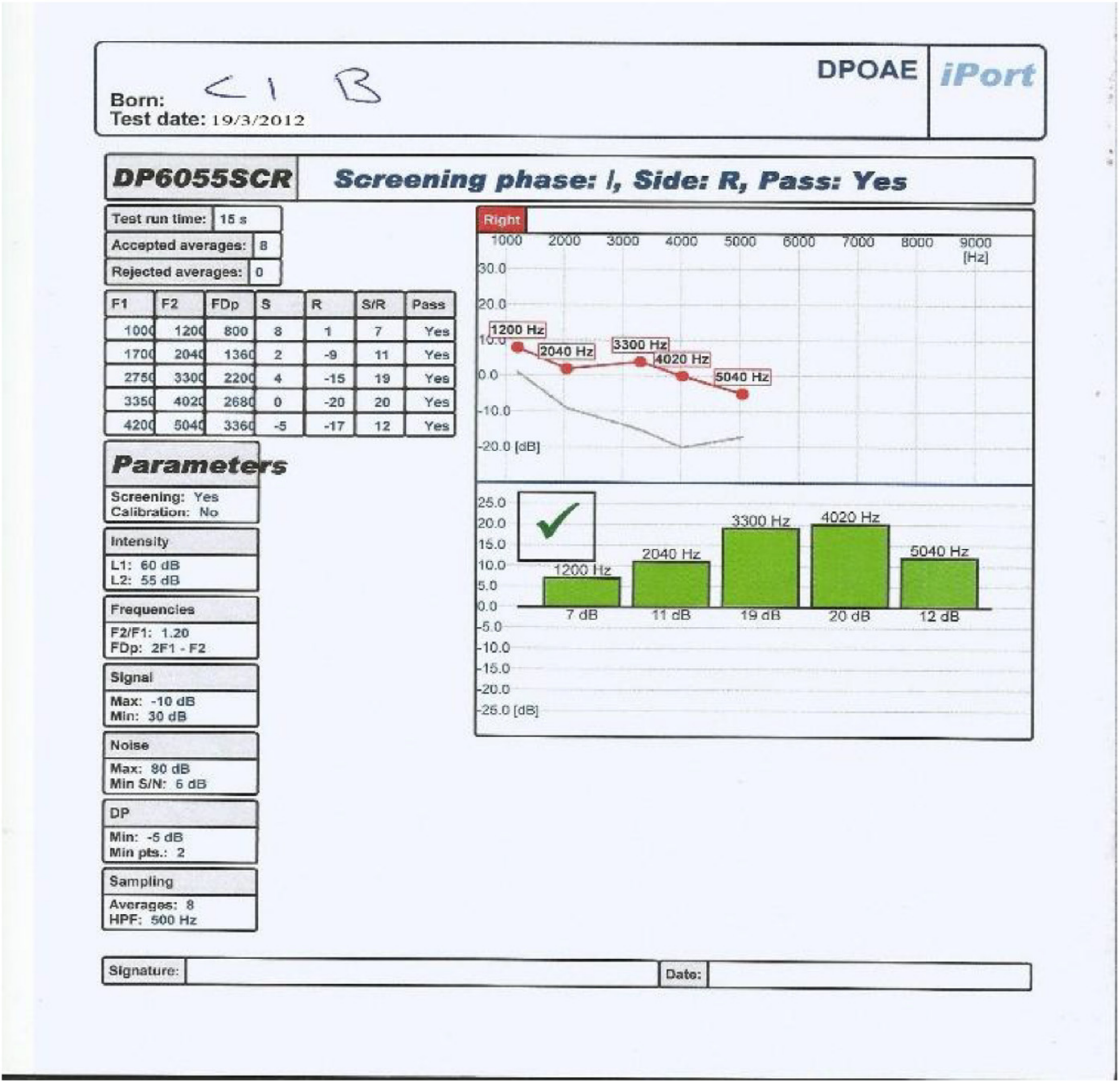
DPOAE of right ear after treatment.

3. Tympanometry: test of the middle ear function was carried out on each ear of All patients in study groups and people in control group using middle ear analyzer (Tympanometer: Amplaid A756 screening, Italia 2009, S/N MRA 7560913009); patients included in this study showed the findings that revealed no abnormalities which may interfere with the accuracy of DPOAEs.
4. We used an automatically designed OAE device (ECHOLAB; it is EcL 110S0 with EcL p03 software, year 2011, version 1.0.1.236. This device had been produced by LABAT INT for otoacoustic emission, allows us to run TOAE, DPOAE and AABR.) It process the results as shown in Fig. 2, Fig. 3, Fig. 4, Fig. 5: the parameters are the F1, F2, FDp, S(Signal), R (noise Ratio), S/R(Signal/noise Ratio and Pass (result).

The occurrence of an answer of DPOAE at a frequency was considered when the amplitude > or = −10 dB NPS and a signal/noise ratio (S/N) > or = 7dBNPS were observed at that frequency, hence the DPOAE reference value classification considered according to factory setting for ECHOLAB was as follow:

∗ Response of DPOAE is present/partially present if the amplitude is higher than 6 dB in 2–5 frequencies.
∗ Response of DPOAE is absent if the amplitude is lower than 6 dB in 1-0 frequencies.

According to the above criteria, our OAE device (ECHOLAB) automatically give and display the result of the test and classify it as pass (green color) or failed (red color) as can be seen in Fig. 2, Fig. 3, Fig. 4, Fig. 5.

Control group was 25 medical staff in our hospital (n3 = 50 ears) who have no Tinnitus and No SNHL and match in age & gender to study groups.

Recordings were repeated two times to make sure the dependability of the results.

We prescribed for every patient in (study group 1 and study group 2) a five Neurorubine™ Ampoules/Acino which is a combination of B **vitamins** to be injected intramuscularly, one ampoule twice a week, after we explained for them the possible benefits or hazards of such treatment and we achieved an informed consent from each patient. Neurorubine™ Ampules were each ampoule contains, as active ingredients, 100 mg vitamin B1 (thiamine hydrochloride), 100 mg vitamin B6 (pyridoxine hydrochloride), 1000 μg vitamin B12 (cyanocobalamin) in 3 ml of aqueous solution for injection.

Exclusion criteria also limits the age younger than 18 years, because no experience or clinical data available to give Neurorubine™ Ampule before this age.

Only 21 patients from study group 1 were come back again after one month, for follow up and repeat OAE study after using a five Neurorubine™ Ampules (vitamin B1, B6,B12), (4 patients with bilateral ear disorder did not return i.e. n1 became = 36 ears), hence we included only first 21 patients from study group 2 who also come back again after one month to keep the number of patients equal in both study groups (i.e. n2 also became = 36 ears).

The clinical(subjective) response to treatment were recorded according to a suggested scale of response to treatment after one month as:

a. Excellent response: if complete absence of tinnitus or improvement in at least 2 grades according (BAOL-HNS).
b. Medium response: if improvement in one grade or more according to (BAOL-HNS).
c. Poor response: if there was no any improvement.

The OAE study repeated and recoded again using the same OAE device (ECHOLAB) after using a five Neurorubine™ ampules (i.e. after one months of first visit). Hence the result of patients with clinical (subjective) response were documented, results of patients DPOAE changes were recorded(objective), results of patients with both clinical response + DPOAE changes (subjective + objective) also recorded.

### 2.2 Statistical analysis

The results processed and analyzed by using MINITAB-14 for both descriptive and inferential statistics.

1. Descriptive Statistics: Statistical tables and percentages.
2. Inferential Data Analysis: T-test used to test two variables, and One –way Anova F-Test.

The level of significance was accepted at P ≤ 0.0 as highly significance(HS), at P < 0.01 as a significant (S), and non-significance at P > 0.5.

This study had been submitted with Research Registration Unique Identifying Number (UIN) at www.researchregistry.com with the number (researchregistry4340).

The work has been reported in line with the STROCSS criteria [24].

## 3 Results

Both study group 1& 2 had been classified demographically in to age group categories, sex categories and whether they are living in urban or rural areas, and the results were found as in Table 1.

**Table 1:** Demographic characteristics of the selected patients with study group 1 & 2.

demographic characteristics	Age group	Age group	Age group	Age group	Gender Male(M)/Femal(F)	Living sit Arban (A)/Rural(R)
	20 y-39 y	40 y-59 y	60 y- 79y	80 y- 100y		
Study group 1	6	9	8	2	20 M/5 F	22 A/3 R
Study group 2	4	12	7	2	18 M/7 F	19 A/6 R

It has been found that among Study group 1. (patients had Tinnitus only), Study group 2. (patients had Tinnitus + SNHL) and the control group: a 16 patients (44%), P-value = 0.000 HS; 28 patients(78%) P-value = 0.000 HS; and 0% respectively, they had decreased Amplitude of DPOAE recorded by OAE device (ECHOLAB) as shown in Table 2.

**Table 2:** The percentage of DPOAE Low Amplitude among Study groups & control.

Groups	Number of Ears	PTA results	DPOAE Low Amplitude	Percent. %	P-value
Study group 1 (Tinnitus pt.)	N1 = 36 (15Bilat.+6 Unilateral	normal	16	44%	0.000 HS
Study group2 (Tinnitus + SNHL pt.)	N2 = 36 (15Bilat.+6 Unilateral)	SNHL	28	78%	0.000 HS
Control group	N3 = 50 (25 bilateral.)	Normal	0	0%	

After one month from the 1st clinical examination and test of the patients by Echolab OAE Device, patients developed a new changes(increase Amplitude of DPOAE) in OAE record among Study group1, study group 2 (whom received Neurorubine™ injection Ampules) and people in control group(whom did not receive Neurorubine™ injection Ampules), the results recorded were: 14 patients(39%) P-value = 0.000 highly significant (HS), 8 patients(22%) P-value 0.026 significant(S), and two persons (5%) P-value 0.88 non-significant (NS) respectively.

Patients whom gave a subjective clinical improvement in their tinnitus after treatment with Neurorubine™ injection Ampules among study group 1 were 14 patients 39%P-value = 0.000 HS, (4 patients showed excellent improvement and 10 patients showed moderate improvement).

In study group 2 they were 6 patients(moderate improvement) 16% P-value = 0.791 NS, and there was no changes(zero) among control group.

Those got (subjective) clinical improvement + increase Amplitude of DPOAE (objective changes) after treatment were 10 patients (28%) in study group1 P-value = 0.000 HS, and two patients (5%) in study group 2 P-value o.321 NS, and there was no changes(zero) among control group after one month (treatment for Study group 1& 2 only) as shown in Table 3, Table 4below.

**Table 3:** After one month (treatment for Study group 1& 2 only).

Groups	increase Amplitude of DPOAE (changes) After treatment		Clinical. Improvement After treatment		Clinical improvement + increase Amplitude of DPOAE (changes) After treatment	
	No.	%	No.	%	No.	%
Study group1. Tinnitus patient.	14	39%	14	39%	10	28%
			4 excellent			
			10 moderate			
Study group2 (Tinnitus + SNHL patient).	8	22%	6 moderate	16%	2	5%
Control group (N0 Treatment)	2	5%	0	0%	0	0%

**Table 4:** One –way Anova F-Test. After one month (treatment for group 1&2).

Groups	increase DPOAE Amplitude		Clinical improvement		Clinical improvement & Increased DPOAE Amplitude	
	F-Test	P-Value	F-Test	P-Value	F-Test	P-Value
Study group1	5.98	0.000 HS	11.36	0.000 HS	12.99	0.000 HS
Study group2	2.55	0.026 S	0.24	0.791 NS	1.18	o.321 NS
Control group	0.12	0.88 NS	0.02	0.99 NS	0.02	0.99 NS

T-test was done to study the relations between the groups, hence there was a significant difference in (DPOAE Decreased Amplitude), i.e. before starting treatment, between Study group1 & Study group2.

The (increase DPOAE Amplitude) after one month from treatment showed high significant difference between Study group1 & Study group2 P-value 0.001 HS. The Studying of the (Clinical improvement) between Study group1 & Study group2 showed No significant statistical difference P-value = 0.138 NS.While the (Clinical improvement & Increased DPOAE Amplitude) between Study group1 & Study group2 appear significant statistically 0.016 (S). while in comparison to control group we found the (Clinical improvement + Increased DPOAE Amplitude) between Study group1 & control group appear high significant difference statistically P-value = 0.000 HS, from other hand Study group2 to control group was P-value = 0.321(NS) as can be seen clearly in Table 5.

**Table 5:** T-Test. relations between the groups before and after treatment.

Groups	T-Test	P-Value
(DPOAE Low Amplitude) Study group1* control group	5.32	0.000 HS
(DPOAE Low Amplitude) Study group2* control group	5.86	0.000 HS
(DPOAE Low Amplitude) Study group1 * Study group2	2.12	0.041 S
(increase DPOAE Amplitude) Study group1 * Study group2	3.61	0.001 HS
(Clinical improvement) Study group1 * Study group2	1.52	0.138 NS
(Clinical improvement & Increased DPOAE Amplitude) Study group1 * Study group2	2.53	0.016 S
(Clinical improvement & Increased DPOAE Amplitude) Study group1 * control group	5.30	0.000 HS
(Clinical improvement & Increased DPOAE Amplitude) Study group2 * control group	1.24	o.321 NS

## 4 Discussion

The results of this study revealed that there was a significant decrease 44% in the DPOAE amplitude in patients among study group 1(tinnitus only) consistent with the results obtained by Hussein Qasem et al. [25], Shiomi et al. [26] Igna et al. [27], Fvaero et al. [28], Vicky et al. [29], and Granjeiro et al. [30] that there was a significant decrease in DPOAEs amplitudes over a limited frequency range observed in the normal hearing tinnitus group when compared to the control group (non-tinnitus normal hearing group).

In contrast, our results do not agree with the results obtained by Gouveris et al. [31] stating that tinnitus ears exhibited relatively increased amplitude of DPOAEs at higher frequencies (4–6.3 kHz) when compared with the group of healthy ears and relatively decreased DPOAEs amplitudes at middle frequencies. The discrepancy between our results and Gouveris et al. results may be due to the availability of an acute progressive lesion of the cochlea such as recruitment “which is the abnormal growth of loudness as the intensity of sound increased”, given that all in Gouveris et al.’s study group had acute symptoms, or could be that the increased amplitude of the DPOAEs in this exact frequency area seems from the fact that most of their patients apparent tinnitus at the 4–6 kHz frequencies and hence that a primary lesion of the cochlea occurs at this specific section, or any injury to the inner hair cells of the cochlea may result in the improved DPOAEs amplitudes [30].

Our study also showed that among Study group 2. (patients had Tinnitus + SNHL) 28 patients(78%) P-value = 0.000 HS had decreased Amplitude of DPOAE recorded by OAE device (ECHOLAB) and this agreed with results seen in the papers of Monique Antunes de Souza Chelminski Barreto, et al. who stated that the statistical analysis revealed that both group evaluated after exposure to impulse noise showed a decrease in amplitudes in comparison with the tests before exposure. From that we can understand that DPOAE test is a sensitive tool to detect subtle shifts after exposure to impulse noise. This demonstrated the applicability of the DPOAE test in monitoring the hearing of soldiers exposed to impulse noise [32].

After one moth from treatment with Neurobine ampules we found that there were a significant changes among patients of study group 1, where they show increase DPOAE Amplitude (P-Value = 0.026 S), Clinical improvement (P-Value = 0.000HS), and (Clinical improvement + Increased DPOAE Amplitude) were also (P-Value = 0.000 HS) this may tell us that the objective indicators by DPOAE changes goes with the clinical improvement indicator (i.e. the percentage of both objective and subject indicators)showed changes after treatment with Neurobine ampules.

That was not the same with group 2 where we got (P-value 0.000HS) for increase DPOAE Amplitude which is an objective indicator, but we got a non-significant changes for both Clinical improvement alone (P-value = 0.791 NS) and also clinical improvement & Increased DPOAE (P-value = 0.321 NS).

Comparing group 1 to group 2 in the name of (Clinical improvement + Increased DPOAE) which is the subjective and objective indicators showed a significant difference (P-value = 0.016 S) for group 1. This may indicate that using Neurobine ampules for tinnitus will be effectively change its severity in patients whom have only tinnitus but may not in patients with SNHL.

Although there were many papers studied the effect of deficiency in vitamins B-complex which has been shown to result in tinnitus and their supplementation may improve the symptom [[7], [8], [9], [10], [11], [12], [13], [14], [15], [16], [17]], Karli R et.al during the work on effect of vitamin B12 deficiency on otoacoustic emissions state that "there appears to be a correlation between vitamin B12 deficiency and cochlear function, hence level of vitamin B12 in the blood should not be ignored in assessment of auditory function" [33] but eventhough we could not find up to our knowledge a paper that compare between the patients with tinnitus only and those with SNHL& tinnitus in the name of (Clinical improvement + Increased DPOAE) after empirical treatment with vitamins B-complex, however our result goes with what Micheal Seidman et al. who stated that some patients have noted that vitamin B1 supplements relieve their tinnitus [9], and also it was the similar results when compare it with pilot study published at 2016 by Singh, Charu who demonstrated that “the significant prevalence of Vitamin B12 deficiency in North Indian population and improvement in tinnitus severity scores and visual analog scale (VAS) in cobalamin-deficient patients receiving intramuscular Vitamin B12 weekly for 6 weeks further provides a link between cobalamin deficiency and tinnitus thereby suggestive of a therapeutic role of B12 in cobalamin-deficient patients of tinnitus'’ [34]. But this was not the case with what Coelho C et al. who said that dietary supplements to treat tinnitus are generally not effective, and many produced adverse effects, hence supplements should not be recommended to treat tinnitus but could have a positive outcome on tinnitus reactions in some people [16].

## 5 Conclusions

From all the above we can suggest that the uses of DPOAE changing amplitude could be used as a parameter to estimate effect of vitamin B complex on changing the severity of tinnitus in patients with tinnitus only and those have Tinnitus with SNHL from other hand the supplementation of vitamin B complex could improve the tinnitus severity especially in patients with tinnitus without SNHL.

As recommendations for this study we can say:

1. More researches are needed in this field with longer time fellow up to estimate effect of vitamin B complex on changing the severity of Tinnitus in patients with tinnitus only and those have Tinnitus with SNHL
2. We recommend that routine B-complex vitamins serum levels be determined whenever it is possible during evaluating patients for tinnitus and those with SNHL.

## Ethical approval

By Ethical committee Approval of the College of Medicine Wasit University under the Judgement's reference number: 34/W 256.

## Sources of funding

No any sources of funding for our research.

## Author contribution

***Dr.Hussam M .Hameed***:Participated substantially in conception, design of the study, medical examination and treatment of patients, collection of data,pre- & post-therapy check of the results.

**Dr. Alaa Husain Eleue, M.D:** Participated substantially in conception, design of the study, collection of data pre- & post-therapy check of the results.

**Dr. Ahmed Mohammed Taqi Al Mosawi**: Participated substantially in conception, design of the study, pre- & post-therapy check of the paper work result.

## Conflicts of interest

All authors have no conflict of interests.

## Trial registry number

It is cohort study.

## Research Registration Number

researchregistry4340.

## Guarantor

Dr.Husam M.Hameed.

## Provenance and peer review

Not commissioned, externally peer reviewed.
